# Changes in pH and Nitrite Nitrogen Induces an Imbalance in the Oxidative Defenses of the Spotted Babylon (*Babylonia areolata*)

**DOI:** 10.3390/antiox12091659

**Published:** 2023-08-23

**Authors:** Ruixia Ding, Rui Yang, Zhengyi Fu, Wang Zhao, Minghao Li, Gang Yu, Zhenhua Ma, Humin Zong

**Affiliations:** 1 Key Laboratory of Efficient Utilization and Processing of Marine Fishery Resources of Hainan Province, Sanya Tropical Fisheries Research Institute, Sanya 572018, China; drxia1021@163.com (R.D.); janeyhn4321@yeah.net (R.Y.); fu0174@flinders.edu.au (Z.F.); zhaowang522@163.com (W.Z.);; 2 South China Sea Fisheries Research Institute, Chinese Academy of Fishery Sciences, Guangzhou 510300, China; 3 College of Science and Engineering, Flinders University, Adelaide 5001, Australia; 4 National Marine Environmental Center, Dalian 116023, China

**Keywords:** gastrapods, immunase, behavior, acidity, alkalinity, nitrous acid

## Abstract

In order to reveal the acute toxicity and physiological changes of the spotted babylon (*Babylonia areolata*) in response to environmental manipulation, the spotted babylon was exposed to three pH levels (7.0, 8.0 and 9.0) of seawater and four concentrations of nitrite nitrogen (0.02, 2.7, 13.5 and 27 mg/L). The activities of six immunoenzymes, superoxide dismutase (SOD), glutathione peroxidase (GSH-PX), catalase (CAT), acid phosphatase (ACP), alkaline phosphatase (AKP) and peroxidase (POD), were measured. The levels of pH and nitrite nitrogen concentrations significantly impacted immunoenzyme activity over time. After the acute stress of pH and nitrite nitrogen, the spotted babylon appeared to be unresponsive to external stimuli, exhibited decreased vigor, slowly climbed the wall, sank to the tank and could not stand upright. As time elapsed, with the extension of time, the spotted babylon showed a trend of increasing and then decreasing ACP, AKP, CAT and SOD activities in order to adapt to the mutated environment and improve its immunity. In contrast, POD and GSH-PX activities showed a decrease followed by an increase with time. This study explored the tolerance range of the spotted babylon to pH, nitrite nitrogen, and time, proving that external stimuli activate the body’s immune response. The body’s immune function has a specific range of adaptation to the environment over time. Once the body’s immune system was insufficient to adapt to this range, the immune system collapsed and the snail gradually died off. This study has discovered the suitable pH and nitrite nitrogen ranges for the culture of the spotted babylon, and provides useful information on the response of the snail’s immune system.

## 1. Introduction

The spotted babylon (*Babylonia areolata*, Link, 1807), commonly known as the ivory shell, belongs to the gastropods. It mainly grows along the coasts of Southeast Asia and Japan, and is a tropical and subtropical marine species, as well as the main aquaculture product and economic shellfish along the southeast coast of China [1]. It is a traditional seafood with rich nutrition and delicious meat, which is favored by people and has a large market capacity [2,3,4]. In recent years, with the breakthrough and upgrade of fry breeding and breeding technology, the industrialized breeding of the spotted babylon has developed rapidly in the southeast coastal areas of China.

The aquatic environment plays an important role in the growth and reproduction of aquatic organisms, while an unsuitable environment can inhibit the development, growth and reproduction of aquatic organisms [5,6]. The factory farming mode has the advantages of environmental protection, control, high efficiency and year-round production. Factory farming of aquatic economic animals is gradually emerging. However, there are problems such as high farming density and overfeeding of bait in the factory-farming process, and there is inevitably a certain concentration of ammonia nitrogen and nitrite nitrogen in the farming water [7,8,9]. Nitrite nitrogen is highly toxic to aquatic animals, and more reports have confirmed that acute nitrite nitrogen exposure can lead to severe histopathological changes and even death in aquatic animals [10]. Nitrite nitrogen is a common pollutant in farmed waterbodies, formed mainly by bacterial nitrosation and denitrification. The increase in human activities, such as the development of high-density farming, the misuse of fertilizers in cultivation and the discharge of industrial wastewater, has intensified nitrite nitrogen accumulation in waterbodies [11,12,13].

pH is an important indicator of the aquaculture–water environment. pH that is too high or too low will directly affect the growth, feeding and metabolism of aquaculture organisms [14]. In industrial shellfish farming, a large number of shellfish deaths, overfeeding of algae and even harmful algal blooms in seawater will lower the pH of the water body [15]. Moreover, during the farming process, many aquatic plants are planted to purify the water, and due to photosynthesis, carbon dioxide (CO_2_) in the water is consumed in large quantities, increasing the pH of the water body. pH changes can cause chronic or acute stress to aquatic organisms and affect their activities and immune functions [16,17,18]. Changes in pH are closely related to the growth and survival of aquatic organisms, and when the pH of the water body deviates from its appropriate range, it will inevitably lead to its survival, feeding, growth and metabolism, ionic balance, and mineralization of the exoskeleton being affected to a certain extent. Therefore, maintaining a suitable and stable pH will help reduce the stress on aquatic organisms and prevent and control diseases.

Studies have shown that maintaining adequate alkalinity concentrations is essential to maintain nitrification [19], and at pH near neutral and alkaline variations in the range of 6.5–9.0, nitrite nitrogen accumulation rises gradually with increasing pH, with pH = 9.0 being more favorable for the accumulation of nitrite nitrogen in denitrification [20]. In the presence of NO, pH has a strong influence on the kinetics and mechanism of abiotic transformation, and low pH conditions tend to easily break S-N and N-C bonds and promote nitrification and nitrosation reactions [21]. The study of chemodenitrification by Fe(II) and nitrite nitrogen, the pH effect, mineralization and kinetic modeling by Chen et al., also found that with the increase in pH, the rate of NO reduction increased, and NO_2_ was produced at the same time [22]. Thus, changes in pH and nitrite nitrogen may become a risk factor for aquatic organisms, not only because tolerance limits are exceeded, but mainly because of possible interactions with chemical or abiotic stressors. With a pH range of 7.8–8.2 and a nitrite range of 0–0.04 mg/L, the spotted babylon culture is divided in two types: indoor cement pond culture and natural sea beach culture [23,24]. The spotted babylon culture density is relatively large, and its main humus-based component is living in the thick sand. The culture process is easy to change the water quality with the spotted babylon feces, dead snails, residual bait, and other large amounts of deposition. Moreover, the natural environment is unstable, and changes in seawater pH and increases in ammonia nitrite nitrogen can be the dominant factors in the environment, which can lead to the slow death of the spotted babylon by massive deposition. These sediments, through the decomposition of microorganisms, produce significant amounts of nitrite nitrogen, ammonia, and other harmful substances, making the substrate seriously blackened and smelly in the process of culture, which, if not resolved in a timely manner, will often cause a large number of deaths of the spotted babylon. Therefore, we should also pay attention to the tolerance of the spotted babylon under natural conditions. This reflects the importance of evaluating the spotted babylon under different water physicochemical parameters. The purpose of this experiment is to evaluate the physiology and biochemistry of the spotted babylon under different water physicochemical parameters and to provide a reference for the healthy, efficient, and sustainable development of the spotted babylon aquaculture industry. The pH values (7.0, 8.0 and 9.0) and nitrite nitrogen concentrations (0.02, 2.7, 13.5 and 27 mg/L) were chosen taking into account the tolerance limits of the spotted babylon [25,26].

## 2. Materials and Methods

### 2.1. Animals

The spotted babylon (*Babylonia areolata,* Link, 1807) was provided by the Tropical Aquatic Research and Development Center of the South China Sea Fisheries Research Institute of the Chinese Academy of Fisheries Sciences (Lingshui County, Hainan Province). The spotted babylon (*Babylonia areolata*) juvenile snails with a body length of (1.70 ± 0.32) cm and a weight of (1.50 ± 0.34) g were used for the experiment and took a 2 d acclimation. Frozen miscellaneous fish were fed daily at 9:00 by satiety. The feces, residual feeds, and the dead spotted babylon were removed from the tank. The water quality parameters during the experimental period were salinity 33.00 ± 0.80, temperature (26.00 ± 1.00) °C, ammonia nitrogen < 0.01 mg/L, nitrite nitrogen < 0.04 mg/L, and dissolved oxygen (DO) > 6.50 mg/L. The natural seawater used in this study was precipitated and sand filtered, as precipitated and sand-filtered seawater purifies the water and is suitable for testing.

### 2.2. Experimental Design

**Figure 1:**
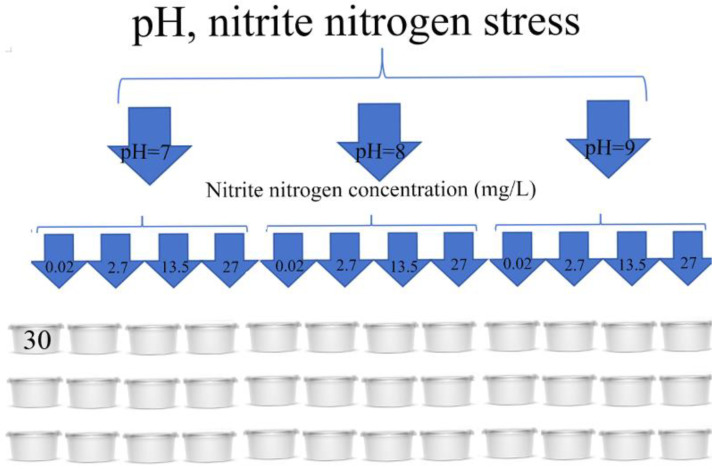
Experimental design of the study.

The 1080 healthy, uniformly sized, and vigorous spotted babylon (30 per tank) were put in 36 tanks (4L). This test was a 3 × 4 test, i.e., the three levels of pH (7.0, 8.0, and 9.0) and four levels of nitrite nitrogen concentrations (0.02, 2.7, 13.5 and 27 mg/L) were set in three parallels for each group (Figure 1). Tanks with pH = 8 and nitrite nitrogen concentrations of 0.02 mg/L were the control group for this experiment. NaNO_2_ was added to seawater to achieve the desired nitrite nitrogen concentration for the test. NaOH and NaHCO_3_ were added to the seawater to achieve the desired pH for the test. The reagents NaNO_2_, NaOH, and NaHCO_3_ were purchased from the Sinopharm Chemical Reagent Co., Ltd. (Shanghai, China). A certain amount of chilled fish is fed at 9:00 every day, and the feeding amount is 6% of the mass of the snail. Water was changed 1 h after feeding, with the exchange rate over 50% of the tank volume each time. The excrement, remaining bait, and dead spotted babylon were removed in time, and the feeding status and activity behavior of the spotted babylons were observed and recorded behavior every 24 h. Seawater pH and nitrite nitrogen were measured and adjusted every 12 h to maintain stable pH and nitrite nitrogen concentrations. The pH was measured by a pH analyzer (SMART SENSOR, Dongguan, China), and nitrite nitrogen concentration was measured by a multiparameter analyzer (Octadem, Wuxi, China).

### 2.3. Behavioral Observation

Behavioral changes in the spotted babylon were observed at 0, 24, 48, 72, and 96 h, respectively. The spotted babylon’s disease was mainly characterized by a slow response to external stimuli, slow movement, reduced wall-climbing, sinking to the bottom of the bucket, and an inability to stand upright. The dead state was that the snail meat was turned out, the anastomosis tube extends outward, and there was whitening and stiffness. The ability to stand indicates that the spotted babylon was less affected by the environment and was in a healthy state. Reversing back proves that the spotted babylon was more affected by the environment and is in an unhealthy state. The reverse-back rate was calculated as follows:

Reverse-back rate (%) = N_t_/N_1_) × 100%

In the equation, N_1_ and N_t_ are the number of snails at the beginning of the test and the behavior observation times, respectively.

### 2.4. Enzyme Activities Analysis

Three random samples were taken from each breeding bucket at 0, 24, 48, 72, and 96 h to detect changes in the enzyme activities. The whole soft tissues were homogenized with 0.2 mol/L normal saline (0.9% NaCl) at a mass-volume ratio of 1:2, and the suspension was centrifuged at 5000 rpm for 10 min at 4 °C. The protein content in the homogenate was determined using the Bradford assay [27]. The activities of Peroxidase (POD; A084-1-1; colorimetric method), Hydrogen peroxidase (CAT; A007-1-1; Ammonium molybdate method), Alkaline phosphatase (AKP; A059-2-2; microenzymatic assay), Acid phosphatase (ACP; A060-2-2; microenzymatic assay), Glutathione peroxidase (GSH-PX; A005-1-2; colorimetric method), and Superoxide dismutase (SOD; A001-3-1; WST-1 method) were determined according to the manufacturer’s instructions using commercial kits (Nanjing Jiancheng Institute of Biological Engineering, Nanjing, China). All parameter analyses were performed in triplicate. Enzyme activities were measured by a multifunctional microplate detector (Synergy H1, Beijing, China) and a UV-visible spectrophotometer (UV-1800BPC, Shanghai, China). Also, the experiment was conducted according to the instructions with negative and positive controls to ensure the validity of the experiment.

### 2.5. Statistical Analysis

The samples in this test were random samples that were independent of each other and conformed to a normal distribution. The data obtained were expressed as mean ± standard deviation (Mean ± SD), and the results were statistically analyzed and tested for homogeneity of variance using SPSS 21.0. The data were first conducted by two-way (3 × 4) analysis of variance (two-way ANOVA), and if there were significant differences between treatments, the means were compared using Duncan’s method at a significance level of *p* < 0.05.

## 3. Results

### 3.1. Effect of pH and Nitrite Nitrogen Stress on the Behavior of the Spotted Babylon

In the pH and nitrite nitrogen stress group, the spotted babylon showed different degrees of stress response with the change of pH and nitrite nitrogen. After pH and nitrite nitrogen stress, the spotted babylon mainly showed sluggish response to external stimuli, slow movement, reduced wall climbing movement, sinking to the bottom of the barrel, and an inability to stand upright. With the extension of time, at the same nitrite nitrogen concentration, compared with the control group (pH = 8.0), the reverse-back rates of pH = 7.0 and pH = 9.0 increased, and the reverse-back rate of pH = 9.0 was higher than the reverse-back rate of pH = 7.0. At the same pH, the reverse-back rate of the spotted babylon increased with increasing nitrite nitrogen concentration. Compared to the control group (pH = 8.0, nitrite nitrogen concentration 0.02 mg/L), the reverse-back rate of the spotted babylon was greater at pH = 9.0 and nitrite nitrogen concentrations of 0.02–27 mg/L than at pH = 7.0 and nitrite nitrogen concentrations of 0.02–27 mg/L over time (Table 1). It can be seen that the effect of pH = 9.0 on the spotted babylon was higher than that of pH = 7.0; the higher the concentration of nitrite nitrogen, the stronger the toxic effect and the higher the reverse-back rate of the spotted babylon; in contrast, the lower the pH and the higher the concentration of nitrite nitrogen, the stronger the toxicity produced by the cross-effects and the more harmful the effect was on the spotted Babylon. Under the same conditions, with the extension of the test time, the reverse-back rate of the spotted babylon was also higher. The lower the pH and the higher the concentration of nitrite nitrogen, the stronger the toxicity caused by its cross-effects, the more harmful it is to the spotted babylon, and the higher its reverse-back rate is; under the same conditions, with the prolongation of the test time, the reverse-back rate of the spotted babylon is also higher.

### 3.2. Effect of pH and Nitrite Nitrogen Stress on GSH-PX Activity of the Spotted Babylon

**Figure 2:**
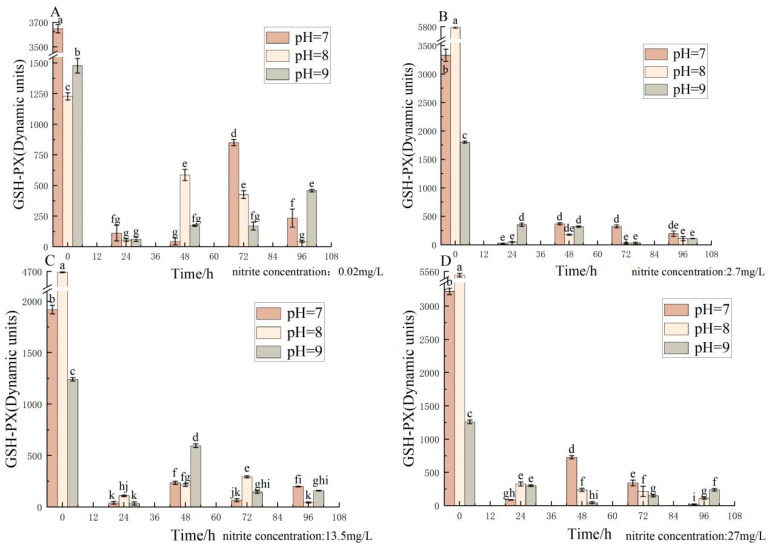
Effect of pH and nitrite nitrogen stress on the GSH-PX activity of the spotted babylon. Orange indicates pH = 7.0; pink indicates pH = 8.0; gray indicates pH = 9.0. (**A**) nitrite nitrogen concentration = 0.02 mg/L; (**B**) nitrite nitrogen concentration = 2.7 mg/L; (**C**) nitrite nitrogen concentration = 13.5 mg/L; (**D**) nitrite nitrogen concentration = 27 mg/L. Significant differences are indicated between different letters (*p* < 0.05).

Different pH, nitrite nitrogen, and pH*nitrite nitrogen treatment times significantly (*p* < 0.05) affected the spotted babylon’s GSH-PX viability (Table 2). The activity of GSH-PX decreased and then increased with time and reached the lowest activity at 24 h, showing an overall decreasing trend. After 24 h, GSH-PX activity at pH 7.0–9.0 did not change significantly at nitrite nitrogen concentrations of 2.7–27 mg/L. GSH-PX activity was significantly lower in the control group (pH = 8, nitrite nitrogen concentration 0.02 mg/L) than in the other groups. GSH-PX activity showed a trend of decreasing and then increasing over time in each test group (Figure 2).

### 3.3. Effect of pH and Nitrite Nitrogen Stress on ACP Activity of the Spotted Babylon

**Figure 3:**
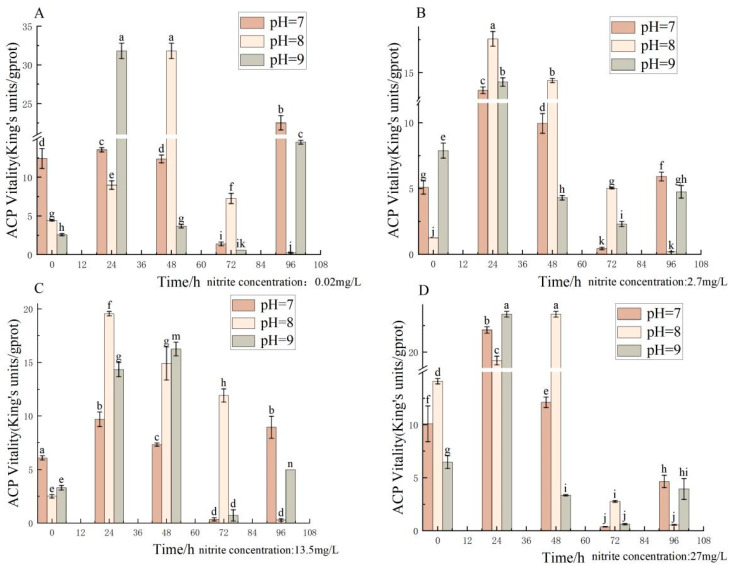
Effect of pH and nitrite nitrogen stress on the ACP activity of the spotted babylon. Orange indicates pH = 7.0; pink indicates pH = 8.0; gray indicates pH = 9.0. (**A**) nitrite nitrogen concentration = 0.02 mg/L; (**B**) nitrite nitrogen concentration = 2.7 mg/L; (**C**) nitrite nitrogen concentration = 13.5 mg/L; (**D**) nitrite nitrogen concentration = 27 mg/L. Significant differences are indicated between different letters (*p* < 0.05).

Different pH, nitrite nitrogen, pH × nitrite nitrogen, and treatment times significantly (*p <* 0.05) affected the spotted babylon’s ACP vigor (Table 3). At pH = 7.0 and nitrite nitrogen concentrations ranging from 0.02–27 mg/L, ACP activity gradually decreased from the highest value at 24 h, after which the overall ACP activity decreased, although it increased over time. At pH = 8.0 and nitrite nitrogen concentrations of 0.02 mg/L and 27 mg/L, ACP activity reached its highest value at 48 h and then gradually decreased. At pH = 9.0 and nitrite nitrogen concentrations of 0.02 mg/L, 2.7 mg/L and 27 mg/L, ACP activity increased to a maximum at 24 h and then decreased significantly with time. Therefore, the ACP activity showed an overall trend of increasing and then decreasing in each treatment group (Figure 3).

### 3.4. Effect of pH and Nitrite Nitrogen Stress on AKP Activity of the Spotted Babylon

**Figure 4:**
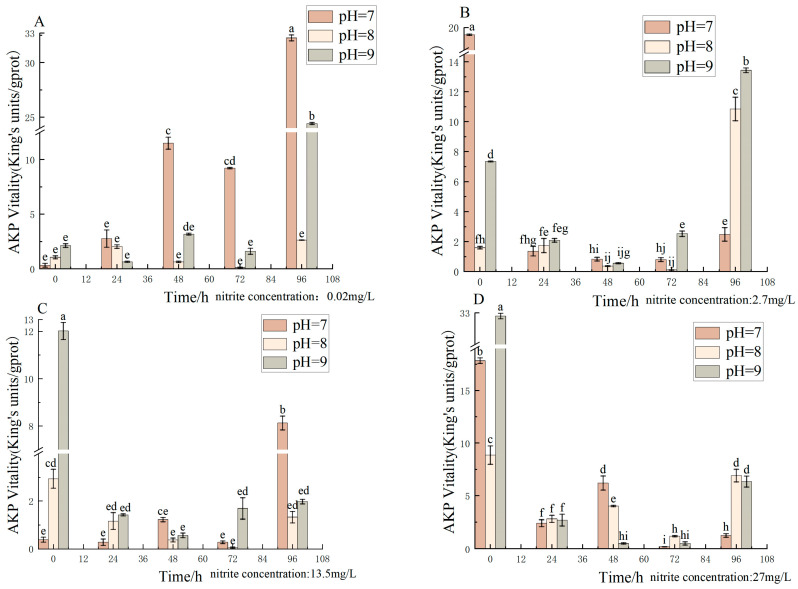
Effect of pH and nitrite nitrogen stress on the AKP activity of the spotted babylon. Orange indicates pH = 7.0; pink indicates pH = 8.0; gray indicates pH = 9.0. (**A**) nitrite nitrogen concentration = 0.02 mg/L; (**B**) nitrite nitrogen concentration = 2.7 mg/L; (**C**) nitrite nitrogen concentration = 13.5 mg/L; (**D**) nitrite nitrogen concentration = 27 mg/L. Significant differences are indicated between different letters (*p* < 0.05).

Different pH, nitrite nitrogen, and pH*nitrite nitrogen treatment times significantly (*p* < 0.05) affected the spotted babylon’s AKP vigor (Table 4). AKP activity did not change significantly at nitrite nitrogen concentrations of 0.02–27 mg/L from 24 h to 72 h when pH was 7.0–9.0. When 0 h, pH = 8.0, 9.0, AKP activity increased with increasing nitrite nitrogen concentration. At pH = 7.0 and nitrite nitrogen concentrations of 0.02–0.27 mg/L, AKP activity was generally increased, although there was no clear linear pattern. Although the pattern of changes in AKP activity varied among the experimental groups, overall it appeared that AKP activity showed a trend of first decreasing and then increasing (Figure 4).

### 3.5. Effect of pH and Nitrite Nitrogen Stress on POD Activity of the Spotted Babylon

**Figure 5:**
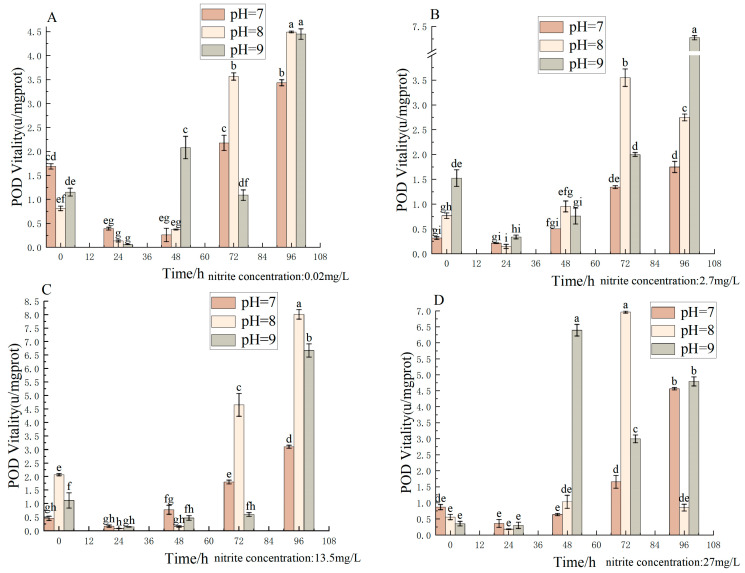
Effect of pH and nitrite nitrogen stress on the POD activity of the spotted babylon. Orange indicates pH = 7.0; pink indicates pH = 8.0; gray indicates pH = 9.0. (**A**) nitrite nitrogen concentration = 0.02 mg/L; (**B**) nitrite nitrogen concentration = 2.7 mg/L; (**C**) nitrite nitrogen concentration = 13.5 mg/L; (**D**) nitrite nitrogen concentration = 27 mg/L. Significant differences are indicated between different letters (*p* < 0.05).

Different pH, nitrite nitrogen, pH × nitrite nitrogen, and treatment times significantly (*p* < 0.05) affected the spotted babylon’s POD vigor (Table 5). At pH 7.0–9.0, the POD activity decreased, increased with time, and decreased to a minimum at 24 h. At pH = 7.0 and 8.0, POD activity showed a significant increase with increasing nitrite nitrogen concentrations (2.7–13.5 mg/L). The most significant change in POD activity was observed at pH 7.0–9.0 and a nitrite nitrogen concentration of 27 mg/L. Overall, POD activity appeared to show a trend of decreasing followed by increasing (Figure 5).

### 3.6. Effect of pH and Nitrite Nitrogen Stress on CAT Activity of the Spotted Babylon

**Figure 6:**
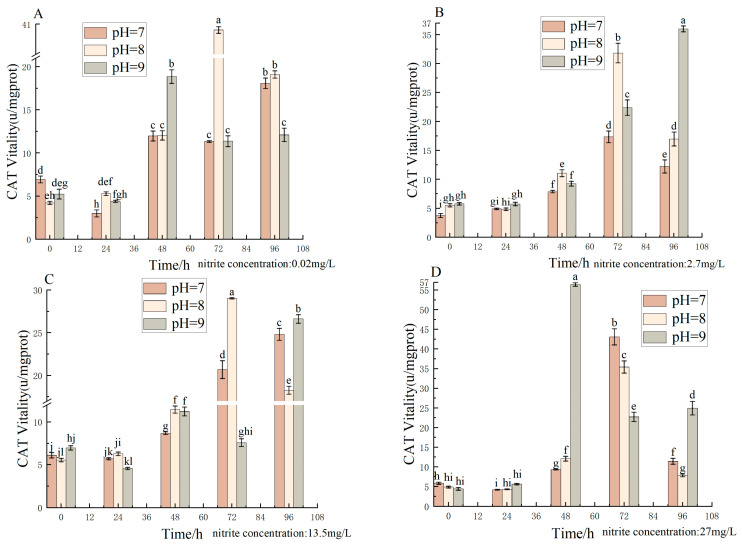
Effect of pH and nitrite nitrogen stress on the CAT activity of the spotted babylon. Orange indicates pH = 7.0; pink indicates pH = 8.0; gray indicates pH = 9.0. (**A**) nitrite nitrogen concentration = 0.02 mg/L; (**B**) nitrite nitrogen concentration = 2.7 mg/L; (**C**) nitrite nitrogen concentration = 13.5 mg/L; (**D**) nitrite nitrogen concentration = 27 mg/L. Significant differences are indicated between different letters (*p* < 0.05).

The effects of different pH, nitrite nitrogen, pH × nitrite nitrogen, and treatment times on the spotted babylon’s CAT vigor were significant (*p* < 0.05) (Table 6). CAT activity did not change significantly at pH (7.0–9.0) and nitrite nitrogen concentrations (0.02–27 mg/L) over a 24 h period. After 48 h and at pH (7.0–8.0) and nitrite nitrogen concentrations (0.02–27 mg/L), the CAT activity gradually increased with the extension of time; the maximum activity appeared at 72 h and then gradually decreased. CAT activity increased with increasing nitrite nitrogen concentrations (2.7–27 mg/L) at pH = 7.0 and 8.0 for the same period of time. CAT vigor varied significantly with a nitrite nitrogen concentration (0.02–27 mg/L) at pH 9. With the increasing time, the CAT activity of each treatment group showed an overall trend of increasing and then decreasing (Figure 6).

### 3.7. Effect of pH and Nitrite Nitrogen Stress on SOD Activity of the Spotted Babylon

**Figure 7:**
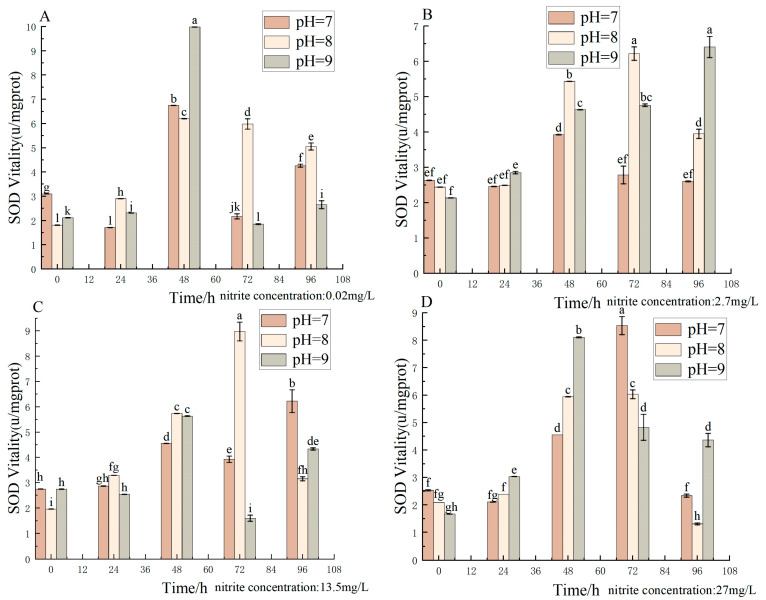
Effect of pH and nitrite nitrogen stress on the SOD activity of the spotted babylon. Orange indicates pH = 7.0; pink indicates pH = 8.0; gray indicates pH = 9.0. (**A**) nitrite nitrogen concentration = 0.02 mg/L; (**B**) nitrite nitrogen concentration = 2.7 mg/L; (**C**) nitrite nitrogen concentration = 13.5 mg/L; (**D**) nitrite nitrogen concentration = 27 mg/L. Significant differences are indicated between different letters (*p* < 0.05).

Different pH, nitrite nitrogen, pH × nitrite nitrogen, and treatment times significantly (*p* < 0.05) affected the spotted babylon’s SOD vigor (Table 7). SOD activity increased with decreasing nitrite nitrogen concentrations (2.7–27 mg/L) at pH = 7.0 and 8.0 for the same period of time. At the same time, SOD activity increased with increasing pH (7.0–9.0) at nitrite nitrogen concentrations of 0.02–0.27 mg/L. Overall, with increasing time, the SOD activity of each treatment group showed a trend of increasing and then decreasing (Figure 7).

## 4. Discussion

### 4.1. Behaviorial Changes of Spotted Babylons

The water environment plays a vital role in the growth and development of aquaculture organisms, and an unsuitable water environment can lead to metabolic stress in aquatic organisms and thus affect their growth. As an important environmental factor, pH and nitrite nitrogen changes may lead to impaired immunity or even the death of aquatic organisms [28,29]. In this study, we found that when nitrite nitrogen was kept at the same concentration, the reversal rate of the spotted babylon at pH = 7.0 and 9.0 was higher than that at pH = 8.0. For example, the reversal back rate of the spotted babylon at pH = 9.0, with a nitrite nitrogen concentration of 27 mg/L, and that of the spotted babylon at pH = 7.0 with a nitrite nitrogen concentration of 0.02 mg/L, were 8 times and 2 times higher than that at pH = 8.0, with a nitrite nitrogen concentration of 0.02 mg/L, respectively. and 0.02 mg/L of nitrite nitrogen were 8 and 2 times higher, respectively. This indicates that pH and nitrite nitrogen interact with each other in the spotted babylon. In other words, the combined effect of pH and nitrite nitrogen would be stronger than pH or nitrite nitrogen alone. Under pH and nitrite nitrogen stress, NO_2_^−^ competes with Cl^−^ and affects the exchange of HCO_3_^−^ with Cl^−^, resulting in an imbalance of ionic balance in the spotted babylon organism [30]. The toxicity of nitrite nitrogen is also manifested in the form of HNO_2_, which can diffuse into cell membranes and, because of its strong oxidizing properties, can destroy the tissue structure and cellular components of spotted babylon, leading to metabolic disorders and a reduction in nonspecific immunity, thus affecting its growth and survival [31]. Therefore, below the pH and nitrite nitrogen stresses, the spotted babylon movement was retarded, the attachment capacity was reduced, and the reverse back appeared.

It has been shown that aquatic organisms can maintain the ionic and acid-base balance of the body by regulating the osmotic pressure through the buffer system in vivo to enable it to adapt to changes in the external environment within a certain range. However, if the buffer limit is exceeded, the ionic and acid-base levels of the body will change significantly, disrupting the ionic and acid-base balance of body fluids and affecting the normal physiological activities of the body [32,33]. The results of this study indicate that the spotted babylon has a degree of tolerance for environmental changes. The results of this study are summarized as follows. At the beginning of the experiment, the spotted babylon survived well and showed no obvious symptoms at pH 7.0–9.0 and NaNO_2_ concentrations of 0.02–27 mg/L. The spotted babylon was also found to have no symptoms. Because the spotted babylon body can still effectively respond to external stimuli in the early stage through the production of antioxidant enzymes to remove superoxide anion in a timely manner, there were still no significant symptoms in the early stage [34]. Changes in ecological factors will induce a series of stress reactions in the organism, in which reactive oxygen radicals generated during the reaction can damage the organism [35]. The enzymatic activities of CAT, SOD and POD decreased with the increase in time and the change in pH (7.0–9.0) and NaNO_2_ concentration (0.02–27 mg/L). This indicates that the spotted babylon’s own antioxidant enzymes can no longer completely scavenge free radicals in the body, which manifests itself as reduced vitality, a slow response to external stimuli, reverse back, etc. It is possible that the cross-talk between pH and nitrite nitrogen affects the tissues and organs of the spotted babylon, e.g., by affecting muscle contractility and neuromediators.

### 4.2. Activity Change of Immunoenzymes

ACP and AKP are two important specific hydrolases with a significant role in eliminating extracellular invaders and are considered sensitive coefficients of the crustacean immune response [36]. ACP is a typical lysosomal enzyme involved in phagocytosis and encapsulation. It plays a role in killing and digesting microbial pathogens during the immune response. At the same time, AKP is an important regulatory enzyme that hydrolyzes phosphate esters and is associated with essential functions in all organisms [37]. *Apostichopus japonicus* had significantly higher ACP and AKP activities under Vibrio splendidus infection [38]. The activity of ACP in the liver and muscle and AKP in the gills of *Oncorhynchus mykiss* was significantly increased during transportation [39]. In this study, we found that the activity of ACP and AKP varied with pH and nitrite nitrogen. For example, the activity of ACP and AKP increased with pH (7.0–9.0) and nitrite (0.02–27 mg/L) compared to the control (pH = 8.0, nitrite concentration 0.02 mg/L).

In the water column, nitrite nitrogen often exists and accumulates in the form of nitrite nitrogen ions (NO^2−^) and nonionic nitrite nitrogen (HNO_2_), of which HNO_2_ is uncharged and can diffuse into the cell membrane. Because of its strong oxidizing properties, it can damage the tissue structure and cellular components of shellfish, leading to metabolic disorders and harming the growth and reproduction of fish and shellfish. pH not only favors the accumulation of nitrite nitrogen, but low pH also promotes the nitrification reaction of nitrite nitrogen, making it more toxic. Numerous studies have shown that pH and nitrite nitrogen can negatively affect the growth and feeding, blood indicators, tissues and organs, and immune functions of aquatic animals, such as fish and shellfish, and also reduce the spawning ability of fish and shellfish, cause changes in hemolymph physiochemical factors and enzyme activity related to disease resistance, and thus reduce defense capacity [40,41]. The oxidative burst (or respiratory burst) is a rapid, short-lived, massive production of reactive oxygen species (ROS), which is one of the important defenses of shellfish against invading microorganisms such as bacteria, fungi, and viruses [42]. However, large accumulations of ROS in the animal’s body can cause severe cellular damage and lead to a variety of diseases. To protect themselves from excessive ROS production, aerobic organisms have produced a series of antioxidant defense systems, including antioxidant enzymes such as CAT, SOD, POD and GSH-PX, which represent the first line of defense for nonspecific immune responses in crustaceans [43]. In this study, the spotted babylon individuals were analyzed for immunoenzymes under pH and nitrite nitrogen stress, and the results showed that the combined effects of pH and nitrite nitrogen resulted in a more intense oxidative stress than pH or nitrite nitrogen alone. It was found that at pH = 7.0–9.0 and a nitrite nitrogen concentration of 0.02–27 mg/L, the overall CAT and SOD activities increased to their maximum value at 72 h and then decreased gradually [44], while the GSH-PX and POD activities showed a trend of decreasing and then increasing [45,46]. The increase in antioxidant-related immunoenzymatic activity may be attributed to the large amount of reactive oxygen radicals produced by the spotted babylon during the initial phase of pH (7.0–9.0), nitrite nitrogen (0.02–27 mg/L), which puts it in a peroxidized state. In order to maintain the dynamic balance of the antioxidant system, the spotted babylon increases the activity of antioxidant enzymes in the body to eliminate the excess reactive oxygen species produced in the body [47,48]. Regarding POD, it is produced by peroxisomes, which are mainly responsible for removing excess H_2_O_2_ produced in the respiratory burst. It was found that POD activity surged after 72 h. The surge in POD activity is most likely to reduce free radical damage in normal cells, thus enhancing the spotted babylon’s immune function and detoxification ability to resist disease infections. However, with the increase in pH (7.0–9.0), nitrite nitrogen concentrations (0.02–27 mg/L), and treatment time, a large amount of reactive oxygen species generated by the body exceeded the scavenging capacity of the antioxidant system, which in turn caused oxidative damage to the cellular structure, and thus the activity of antioxidant enzymes CAT and SOD decreased after 72 h. This finding also supports the results of other shellfish studies, such as those of shrimp exposed to WSSV with decreased SOD levels [49]. Therefore, the large amount of ROS produced by the organism at pH = 7.0, 9.0, and a nitrite concentration of 27 mg/L over time exceeded the scavenging capacity of the antioxidant system, which caused oxidative damage to the cellular structure and reduced the activities of the antioxidant enzymes CAT and SOD. All these data suggest that the ROS system has a significant role in the innate immune defense of shellfish against various pathogens. Therefore, to a certain extent, the time it takes for immune enzyme activities to return to normal levels also reflects the body’s ability to adapt to the environment.

However, the changes in the immune enzyme activities with pH and nitrite nitrogen concentrations were significant but specific, and there was no obvious linear relationship or consistent pattern, which being related to the different developmental stages of the spotted babylon, its immunity or high resistance to stress, or its immune system may be disrupted by the pH and nitrite nitrogen stress. However, the stress treatment measures were still within their adaptation range. At the same time, there are also many studies showing that different sizes of experimental subjects, different culture densities, and different environmental factors, including dissolved oxygen and baiting residues, can also cause significant differences in pH and nitrite nitrogen stress. The results showed an interaction between pH and nitrite nitrogen and an effect on most antioxidant and immune indices. Therefore, further study is required to elucidate the potential mechanism in the spotted babylon.

## 5. Conclusions

In the present study, the combined effects of pH and nitrite nitrogen on the antioxidants and immunity of the spotted babylon were investigated biochemically and proteolytically. The results showed that when pH was 9.0 and the nitrite nitrogen concentration was 27 mg/L, the effect on the spotted babylon was more significant. Oxidative stress was enhanced by a combination of pH and nitrite nitrogen, resulting in the production of excess reactive oxygen species (ROS). The antioxidant enzyme system was activated to scavenge the excess ROS, while the immune system was activated to keep the body in a healthy state. This study provides new insights into the antioxidant and immune mechanisms of the spotted babylon under multiple culture conditions. And the pH and nitrite nitrogen acute stress in the spotted babylon immune enzyme activity changed patterns, which is a good guide to the water environment regulation range. Therefore, when aquatic animals such as the spotted babylon are cultured, certain methods should be adopted to reduce the stress caused by pH and high nitrite nitrogen concentrations, so as to minimize the hazards of pH and nitrite nitrogen to aquatic organisms.

## Figures and Tables

**Table 1:** Effects of pH (7.0, 8.0 and 9.0) and nitrite nitrogen (0.02, 2.7, 13.5 and 27 mg/L) stress on the behavior and reverse-back rate of the eastern snail.

		Reverse-back Rate/%					Behavior				
		0 h	24 h	48 h	72 h	96 h	0 h	24 h	48 h	72 h	96 h
pH = 7.0	0.02 mg/L	0	0	0	0	2 ± 0.6	Normal	Normal	Normal	slow	reverse back
	2.7 mg/L	0	0	0	2 ± 0.2	4 ± 0.2	Normal	Normal	slow	reverse back	reverse back
	13.5 mg/L	0	0	0	4 ± 0.3	6 ± 0.9	Normal	Normal	slow	reverse back	reverse back
	27 mg/L	0	0	0	5 ± 0.9	8 ± 0.4	Normal	Normal	slow	reverse back	reverse back
pH = 8.0	0.02 mg/L	0	0	0	0	0	Normal	Normal	Normal	Normal	slow
	2.7 mg/L	0	0	0	0	1 ± 0.2	Normal	Normal	Normal	slow	reverse back
	13.5 mg/L	0	0	0	0	3 ± 0.2	normal	Normal	Normal	slow	reverse back
	27 mg/L	0	0	0	3 ± 0.2	5 ± 0.1	Normal	Normal	Normal	reverse back	reverse back
pH = 9.0	0.02 mg/L	0	0	0	0	3 ± 0.7	Normal	Normal	Normal	slow	reverse back
	2.7 mg/L	0	0	0	3 ± 0.2	5± 0.1	Normal	Normal	slow	slow	reverse back
	13.5 mg/L	0	0	0	4 ± 0.7	7 ± 1.2	Normal	Normal	slow	reverse back	reverse back
	27 mg/L	0	0	0	6 ± 0.9	9 ± 1.3	Normal	Normal	slow	reverse back	reverse back

Note: Reverse back means the spotted Babylon with the ventral side up and the shell down in an unhealthy state.

**Table 2:** Multivariate ANOVA of the effects of pH and nitrite nitrogen stress on the activity of the spotted babylon GSH-PX.

Source	df	Mean Square	F	Sig.
Corrected Model	58	5,377,369.576	791.063	0.001
Intercept	1	99,742,175.04	14,673.039	0.001
Time	4	53,430,113.16	7860.087	0.001
pH	2	4,996,513.484	735.035	0.001
NaNO_2_	3	591,197.699	86.971	0.001
Time × pH	8	5,024,023.875	739.083	0.001
Time × NaNO_2_	12	1,013,173.161	149.048	0.001
pH × NaNO_2_	6	1,146,681.894	168.688	0.001
Time × pH × NaNO_2_	23	1,246,782.462	183.414	0.001
Error	121	6797.649		

**Table 3:** Multivariate ANOVA of the effects of pH and nitrite nitrogen stress on the activity of the spotted babylon ACP.

Source	df	Mean Square	F	Sig.
Corrected Model	58	148.324	183.276	0.001
Intercept	1	11,255.171	13,907.373	0.001
Time	4	1251.402	1546.286	0.001
pH	2	66.525	82.202	0.001
NaNO_2_	3	58.397	72.158	0.001
Time × pH	8	56.297	69.563	0.001
Time × NaNO_2_	12	82.259	101.642	0.001
pH × NaNO_2_	6	64.737	79.992	0.001
Time × pH × NaNO_2_	23	60.275	74.478	0.001
Error	121	0.809		

**Table 4:** Multivariate ANOVA of the effects of pH and nitrite nitrogen stress on the activity of the spotted babylon AKP.

Source	df	Mean Square	F	Sig.
Corrected Model	58	553.398	131.221	0.001
Intercept	1	7056.206	1673.155	0.001
Time	4	1410.927	334.557	0.001
pH	2	670.532	158.995	0.001
NaNO_2_	3	697.56	165.404	0.001
Time × pH	8	195.971	46.468	0.001
Time × NaNO_2_	12	969.969	229.997	0.001
Ph × NaNO_2_	6	319.657	75.796	0.001
Time × pH × NaNO_2_	23	354.439	84.044	0.001
Error	121	4.217		

**Table 5:** Multivariate ANOVA of the effects of pH and nitrite nitrogen stress on the activity of the spotted babylon POD.

Source	df	Mean Square	F	Sig.
Corrected Model	58	12.96	61.064	0.001
Intercept	1	623.051	2935.726	0.001
Time	4	96.425	454.34	0.001
pH	2	14.306	67.406	0.001
NaNO_2_	3	2.291	10.793	0.001
Time × pH	8	11.654	54.913	0.001
Time × NaNO_2_	12	6.459	30.432	0.001
pH × NaNO_2_	6	4.465	21.039	0.001
Time × pH × NaNO_2_	23	5.4	25.446	0.001
Error	121	0.212		

**Table 6:** Multivariate ANOVA of the effects of pH and nitrite nitrogen stress on the activity of the spotted babylon CAT.

Source	df	Mean Square	F	Sig.
Corrected Model	58	394.751	432.259	0.001
Intercept	1	34,471.143	37,746.486	0.001
Time	4	2721.773	2980.388	0.001
pH	2	251.208	275.078	0.001
NaNO_2_	3	244.356	267.574	0.001
Time × pH	8	311.775	341.398	0.001
Time × NaNO_2_	12	256.935	281.348	0.001
pH × NaNO_2_	6	268.576	294.096	0.001
Time × pH × NaNO_2_	23	156.744	171.637	0.001
Error	121	0.913		

**Table 7:** Multivariate ANOVA of the effects of pH and nitrite nitrogen stress on the activity of the spotted babylon SOD.

Source	df	Mean Square	F	Sig.
Corrected Model	58	41.465	491.993	0.001
Intercept	1	3315.377	39,337.969	0.001
Time	4	169.983	2016.898	0.001
pH	2	37.784	448.323	0.001
NaNO_2_	3	32.664	387.568	0.001
Time × pH	8	40.713	483.067	0.001
Time × NaNO_2_	12	33.487	397.338	0.001
pH × NaNO_2_	6	43.742	519.018	0.001
Time × pH × NaNO_2_	23	26.555	315.08	0.001
Error	121	0.084		

## Data Availability

For all articles published in MDPI journals, copyright is retained by the authors. Articles are licensed under an open access Creative Commons CC BY 4.0 license, meaning that anyone may download and read the paper for free. In addition, the article may be reused and quoted provided that the original published version is cited. These conditions allow for maximum use and exposure of the work, while ensuring that the authors receive proper credit.
